# Monitoring system of implementation of the Promoting Mental Health at Schools (PROMEHS) program

**DOI:** 10.3389/fpsyg.2022.1043001

**Published:** 2022-10-26

**Authors:** Baiba Martinsone, Ieva Stokenberga, Ilaria Grazzani

**Affiliations:** ^1^Department of Psychology, University of Latvia, Riga, Latvia; ^2^Department of Human Sciences for Education “R. Massa”, University of Milano-Bicocca, Milan, Italy

**Keywords:** monitoring, social-emotional learning, mental health, fidelity, dosage, quality, responsiveness, adaptation

## Abstract

Effective school-based mental health programs are a research field with growing interest and great social value. At the stage of development and initial testing of the program, as well as during dissemination, and adaptation in other cultures, it is important that the implementation is carried out in the way that was originally intended. Fidelity or adherence is the most often used concept relating to the extent to which the implemented intervention corresponds to the originally intended program. Therefore, monitoring of the implementation is an essential element necessary to integrate into contemporary evidence-based program. The current paper describes the monitoring system developed for the Promoting Mental Health at Schools (PROMEHS) project. The monitoring was done on both the structural and procedural aspects of the program’s implementation, involving the evaluation of five core aspects: fidelity, dosage, quality, responsiveness, and adaptation. This methods article aims to describe the development of the monitoring system and to analyze the strengths of the qualitative-quantitative multi-informant approach in the monitoring of the intervention’s implementation. In the future, this would support further research on effectiveness of the PROMEHS program.

## Introduction

### Monitoring as a key aspect of qualitative/reliable program implementation

Evidence connecting school-based mental health program outcomes with implementation components are increased rapidly during last years, especially in the US (Rojas-Andrade and Bahamondes, 2019). It was supported by growing body of the scientific studies of the implementation field and following recommendation for testing, implementing, and disseminating evidence-based programs (Domitrovich et al., 2008; Proctor et al., 2013). Several models guiding implementation and monitoring of the implementation fidelity are prevalent in the literature, demonstrating broad scope and variability of components (Fixsen et al., 2021). Focus on defining usable innovation (active components hypothesized to cause effect) and implementations drivers (actors) and stages (procedure) characterize majority of them.

Factors in macro-level, school-level, and individual level can affect successful program implementation in schools (Domitrovich et al., 2008). Several of them has been recognized in the literature and proved to be crucial for the school-based mental health interventions (e.g., teacher competence and support from the head of the school) (Lendrum et al., 2013). Among the factors depending on the implementation process, there are several that should be emphasized: The support system of program providers (i.e., training and assistance during the implementation), compatibility of the innovation, providers’ attitudes and beliefs, community resources, and general and specific organizational factors (e.g., Stith et al., 2006; Durlak and DuPre, 2008; Wandersman et al., 2008). Teachers’ positive attitude toward the program and understanding of the core components is crucial because it allows to make them necessary adaptation without negative cost for quality and predicts fidelity of the program implementation in the long term (Sørlie, 2021).

It is known that the implementation process is related to the outcomes of programs when their effectiveness is evaluated (e.g., DuBois et al., 2002; Wilson et al., 2003; Durlak et al., 2011). Moreover, faithful replication is even more important when programs are disseminated to use in the field, where development and testing of the program is not the focus. Even a well-developed program could become less effective or even ineffective over time without proper dissemination, introducing it to the potential implementers, support for the acceptance of the program, and investment in its sustainability. Recent study in Norway supports necessity to start implementation monitoring in the early stages of the intervention, because these data predict fidelity of the program in the long term (Sørlie, 2021). Thus, the validity of an intervention should be ensured by consistent monitoring of the implementation process.

There are several implementation components important for monitoring described in the literature (Durlak and DuPre, 2008; Durlak, 2015). The criteria relating to a program’s implementation are fidelity (correspondence of the implemented program to the originally intended one), dosage (quantity of delivered content of the intervention), quality (how well the program has been conducted), and the responsiveness of participants. Some authors also note the differentiation between or the extent to which the content and methodology of a program are distinct from other programs as a considerable aspect (Dane and Schneider, 1998; Durlak, 2015). In the recent literature (e.g., Mohr et al., 2014) the necessity to monitor the control group, participation rate, and the representativeness of groups involved, as well as the extent of adaptations to or modifications of the program during the implementation process is also emphasized.

Several components should be included in the monitoring because we do not know which are the most important implementation factors. In previous studies, different components have been found to be the most significant implementation factors. It has been proved that interventions implemented in high fidelity show stronger effect on outcome (Durlak et al., 2011). Recent analysis found that students’ exposure (number of classes) and receptiveness (student commitment) are among those with the strongest impact (Rojas-Andrade and Bahamondes, 2019).

In most cases, only few components have been assessed during the monitoring of the implementation of different preventive programs (for a review, see Durlak and DuPre, 2008; Rojas-Andrade and Bahamondes, 2019). Fidelity and dosage are implementation components included in the studies most often, and typically measured quantitatively using self-report data. Responsiveness, in contrast, needs observational data from several informants as commitment to the program is crucial for both instructors and participants.

Durlak (2015) emphasizes that it is not possible to avoid adaptation in field studies and following dissemination. Some of the modifications can be beneficial (e.g., adding culturally relevant material contributing main program aim), but some – negative (e.g., selecting only certain type of activities or shortening the time of activity). It is crucial to document adaptations made during implementations, and to evaluate their value according the aims of the program and implementation context. Moreover, adaptation is typically measured qualitatively, allowing to provide more contextualized information about the implementation.

There are several highly valuable examples when psychometrically sound measures of fidelity are developed for certain programs (e.g., Abry et al., 2015), or intervention systems as School-Wide Positive Behavior Support (Horner et al., 2004). For example, in Norwegian PBIS program (Sørlie et al., 2015) implementation dosage was estimated by percentage of trained school staff, and quality of the implementation has been measured by asking teachers how do they implement support to positive behavior (e.g., “Expected student behavior is consequently encouraged and positively acknowledged”). This teacher behavior addresses one of the core component of the program, and scale composed from several items is useful for self-report or observation. Considering core components of each program procedures and measures should be developed for the monitoring of the implementation process both for faithful replication and evaluation of the possible effect on outcomes.

Nevertheless, evaluating the implementation of a wide spectrum of preventive and intervention programs provides empirical evidence on the key role of appropriate implementation in the success of programs. These factors were considered when the monitoring system of the Promoting Mental Health at Schools (PROMEHS) program was developed.

### The Promoting Mental Health at Schools program

The Promoting Mental Health at Schools was developed within the Erasmus + Key Action 3 project co-funded by the European Commission. The project’s timeline was from 2019 to August 2022, and it aimed to develop a comprehensive mental health curriculum, implement it, and evaluate its effectiveness. The consortium involved researchers, practitioners, and policymakers from seven European countries: Italy, Latvia, Portugal, Croatia, Romania, Greece, and Malta.

The PROMEHS theoretical framework includes three domains, namely, promoting social-emotional learning (SEL) and resilience and preventing social, emotional, and behavioral problems. This framework was described and substantiated by Cavioni et al. (2020).

The key features of the universal curriculum were based on principles of international research (CASEL, 2020), such as the whole-school approach, evidence-based content, multi-year handbooks, developmental perspectives, teacher training, etc. The capacity of this curriculum was built through teacher training and ongoing assistance, sustaining partnerships with policymakers, and parents’ involvement.

The PROMEHS curriculum consists of seven handbooks. Two are for teachers with ready-to-use, step-by-step activity plans for leading pre-school and school students aged from 3 to 18 years. Two handbooks are for both pre-school/primary school students and middle/secondary school students with activities to carry out independently at home or together with their parents. The other three handbooks are for teachers to promote their own mental health, for parents to promote mental health at home, and for supplying recommendations to policymakers.

Since the curriculum was aimed at fostering students’ SEL and resilience and preventing social, emotional, and behavioral problems, all these topics were covered in the offered activities. Each activity has the same structure, namely, defined learning outcomes, a clearly defined age group, and a step-by-step activity plan. The activity starts with a story, followed by a discussion, role-play, group work, or another learning strategy. An important part of the activities is reflection. At the end of every activity, a teacher is provided with a brief formative evaluation chart, tips on how to embed the goal into their everyday teaching practices, as well as culturally adapted further resources (lists of books, movies, videos).

The curriculum was implemented in Italy, Latvia, Portugal, Croatia, Romania, and Greece, whereas the University of Malta acted as the external evaluator and was not involved in the development and implementation of PROMEHS. The project’s implementation and the evaluation of its effectiveness were carried out in four age groups of students from pre-school to secondary school level (3–6, 8–10, 11–13, and 14–16 years), including disadvantaged children.

**FIGURE 1:**
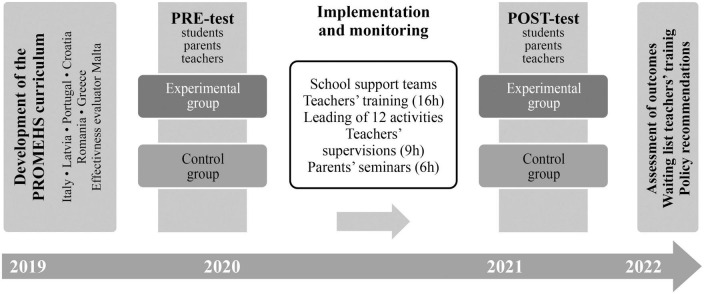
Promoting Mental Health at Schools project’s timeline and design.

The quasi-experimental research design with experimental and control conditions was implemented to evaluate the effectiveness of the program. An integral part of the development and implementation of the PROMEHS program was the monitoring system, which was built with the purposes of ensuring the fidelity and quality of its implementation and of finding out culture-specific practices to develop recommendations for practitioners and educational policymakers (see Figure 1). A detailed description of the curriculum and the whole project is available in Cefai et al., 2022a,b.

### Framework of the Promoting Mental Health at Schools implementation monitoring system

The monitoring of the implementation can be done for diverse purposes, and decisions should always consider the balance between costs and added value. In this case, the purpose of the monitoring, as defined in the project proposal, was to evaluate the quality of the intervention’s implementation (1) to ensure the fidelity and quality of its implementation and (2) to find out culture-specific practices for schools to develop recommendations for both practitioners and educational policymakers.

Five dimensions were used for this purpose: fidelity, dosage, quality, responsiveness, and adaptation (Dane and Schneider, 1998; Durlak and DuPre, 2008; Feely et al., 2018).

Fidelity characterizes the extent to which the implemented intervention corresponds to the originally intended program. The fidelity of the implementation of the PROMEHS program was supported by the provision of detailed materials on the content and procedures to be implemented. Both structural (the content to be delivered) and process components (how the content should be implemented) of the program’s implementation were described in the PROMEHS materials. Comprehensive and detailed handbooks were developed for each age group, both for teachers and students (Grazzani et al., 2020a,b,c,d), teacher training and a series of supervisions were carried out, and activities for school leaders and parents’ meetings were organized in line with the curricula with the aim to increase fidelity or adherence.

Dosage refers to how much of the intervention has been delivered. It has a high potential to be included in effectiveness studies, and therefore it was decided to monitor it as well. In the implementation of the PROMEHS, minimal exposure was defined as 12 activities proportionally covering all three parts of the program, namely SEL, promoting resilience, and preventing behavioral problems.

Quality refers to how well different program components have been implemented. The quality of implementation evaluates the competence of the program providers according to the content and manner of the intervention manual. Organizational factors (e.g., education or qualification requirements) are recognized as a useful way to increase the quality of the intervention. However, its combination with process evaluation is crucial, especially when using external observations (Feely et al., 2018).

Participant responsiveness refers to the degree to which the program stimulates the interest and engagement of participants. Most often, it is the responsiveness of the direct target group (e.g., students) that is measured (Durlak and DuPre, 2008). Considering that the success of the intervention is affected by the involvement of both school and family, a multi-informant approach was used, and all of them–teachers, students, and parents–were treated as participants.

Considering that the model for assessing the fidelity of the PROMEHS project’s implementation was developed to provide information on how its implementation may vary across countries and to provide specific recommendations for its implementation in the future, fidelity is supplemented by adaptation assessments. Adaptation refers to changes made to the original program during its implementation (program modification). Previous research (Durlak and DuPre, 2008; Feely et al., 2018) suggests that adaptation should be evaluated separately (rather than as a failure to achieve fidelity) because it could make possible positive contributions to the outcome(s). Culture-specific adaptations can provide important insight into the best implementation practices crucial for the sustainability of the program (Forman et al., 2009) at the national and international levels.

## Methodology

### Research context and participants

The PROMEHS program was implemented in six European countries in the school year 2020/2021. Initially, it involved 10,209 students, but pre- and post-test evaluations were received from 4,501 participants in the experimental condition and 3,288 participants in the control group, where the evaluators were teachers. Both pre- and post-test parental evaluations were received in relation to the outcomes of 2,394 participants in the experimental group and 2,234 participants in the control group. Student self-reports at the two measure points were obtained from 1,845 students in the experimental group and 1,458 from the control group.

However, monitoring the data collection was not directly related to pre- and post-test data for the effectiveness study. The monitoring sample consisted of experimental condition participants, namely 2,534 students from primary and middle/secondary school (aged nine and older) and 2,868 parents, who provided feedback after their children’s participation in the program activities (See Table 1).

**TABLE 1:** Sample sizes for monitoring the implementation of Promoting Mental Health at Schools (PROMEHS).

Sample size in each informant group by country	Italy	Latvia	Romania	Croatia	Greece	Portugal	Total
School support team members	5	3	5	5	3	8	29
Teachers (training)	192	51	94	64	63	68	532
Teachers (3rd supervision)	140	54	93	34	45	55	421
Parents	296	728	704	273	250	617	2,868
Student (post-test) Primary school Middle and high school	154 236	209 353	235 328	26 55	139 50	346 403	1,109 1,425

During the project’s implementation, 532 teachers were trained in total, of whom 421 filled out the final evaluation of the program, identifying the strengths and weaknesses of the materials and providing practitioners with their expertise for the further elaboration of the PROMEHS materials.

School support teams were organized in each country, with a range of members from three to eight per country. In sum, there were 29 members, all qualified professionals with specific knowledge and expertise as described in the quality requirements. They organized pre- and post-test data collection, managed teacher training and on-going supervisions, collected qualitative data from teachers, and contributed to developing detailed recommendations for the further elaboration of the PROMEHS program’s materials and its implementation in diverse contexts (e.g., remotely).

The number of participants was different between the countries due to different response rate (in groups of students and their parents), and the teachers’ involvement (in some countries, more teachers participated in the PROMEHS than it was planned in the research protocol).

### The monitoring system and measures

Several steps were taken for the development of the monitoring system as recommended in the literature (Feely et al., 2018): (1) defining the purpose and scope of the monitoring; (2) identifying the components for assessment; (3) developing the tools for assessment; (4) collecting data during the project’s implementation; and (5) analyzing the data.

The monitoring system was developed by the first two authors of the paper in collaboration with project partners. Considering the purpose of the monitoring and principles of the program, a multi-component and multi-informant approach was chosen.

Detailed implementation procedures were developed following recommendations in literature (Domitrovich et al., 2008; Proctor et al., 2013; Fixsen et al., 2021). Essential components were identified based on the PROMEHS program and considering the importance of monitoring its implementation in all stages of the field trial, starting with the development of the school support team, providing teacher training and supervisions, followed by providing activities at schools and parents’ meetings, as well as the management of the implementation. This approach was also based on the indicators supporting program sustainability (proposed by Elias, 2010), such as building a support system for teachers involving personnel outside the school’s staff, providing on-going professional development for teachers, as well as integrating the program into the regular curriculum.

Following examples in the previous studies (e.g., Sørlie et al., 2015) and guidelines (e.g., Proctor et al., 2013) indicators for each monitoring dimension were identified, discussed, and selected. Items corresponding to each indicator were developed based on the balance between yes/no, Likert-type scale and open-ended questions. Item formulations were matched with the context in which different informants were expected to respond (e.g., quantitative scales for items about the clarity of the task after the training; open-ended questions for reflections about successes and difficulties experienced after each activity).

All procedures characterizing process components (how the program should be implemented) were discussed with project partners and translated into six national languages. All measures were piloted with the appropriate target audience, tested and corrected for clarity, discussed with partners, and translated into six national languages. Finally, monitoring data were collected during all stages of the field trial and analyzed before the results were presented to project partners.

The Ethics Committee for Humanities and Social Sciences Research Involving Human Participants of the University of Latvia granted permission for the research on 12 December 2019.

## Results

The PROMEHS monitoring system resulting from the procedures and measures developed for monitoring purposes can be seen in Table 2.

**TABLE 2:** Monitoring system for evaluating the implementation of Promoting Mental Health at Schools (PROMEHS).

Program components	Materials and procedures	Monitoring dimensions and indicators	Measures and informant	Informant
School support team	Development of the school support team Sharing procedures and materials for data collection and teacher training	Quality: qualification requirements; competence in teaching materials and procedures Fidelity: consequent implementation of the program components	Supplementary Annex 1. Table for keywords Supplementary Annex 2. Checklist about competence in materials and procedures Supplementary Annex 11. Checklist of activities	School support team members
Teacher training	Curriculum Handbooks for pre-school/primary school Handbooks for middle/high secondary school	Fidelity: 16 h training was organized (time, place, duration, number of participants); adherence to agenda Responsiveness: perceived Teachers’ responsiveness and acceptance of the content	Supplementary Annex 3. Questionnaire of evaluation of teacher training	School support team members
		Quality: perceived usefulness of the training, sufficiency of information, understanding of the task to be performed, confidence in ability to carry out this program Quality: competence in teaching materials	Supplementary Annex 4. Questionnaire of evaluation of teacher training Supplementary Annex 5. Table for keywords	Teachers
Supervisions	Guidelines	Fidelity: 3 × 3 h supervisions were organized (time, place, duration, number of participants) Adaptation: best practices and changes made in the program	Supplementary Annex 7. Supervision summary	School support team members
Activities at schools	Handbooks for pre-school/primary school Handbooks for middle/high secondary school Handbook for teachers	Fidelity: program implemented as described in the manual Dosage: number of activities implemented Quality: observed evidence of students’ competence, perceived effect on self-development in teaching SEL. Responsiveness: teachers’ perception of the students’ responsiveness; usefulness of handbook for teachers	Supplementary Annex 6. Teacher self-reflection form Supplementary Annex 8. Final evaluation form in 3rd supervision	Teachers
		Quality: evaluation of the teaching process Responsiveness: using students’ handbook	Supplementary Annex 9. Student survey	Students
Meeting with school leaders	Guidelines for policymakers	Fidelity: meeting was organized	Supplementary Annex 11. Checklist of activities	School support team members
Parents’ meetings	Curriculum Handbook for parents	Responsiveness: evaluation of the parents’ meetings; evaluation of the handbook for parents and students’ handbook	Supplementary Annex 10. Parent survey	Parents

The PROMEHS monitoring procedures are presented in Table 3.

**TABLE 3:** The Promoting Mental Health at Schools (PROMEHS) monitoring procedures.

(1). *Developing the school support team* Description of competencies of the schools’ support team members: • appropriate qualification, desire to be a psychologist; • do not work in the same school; • familiar with the mental health concept and school environment; • experience of working with groups; • good knowledge of the PROMEHS materials; • understanding of research principles and ethics.
(2). *Sharing procedures and materials of data collection with school support team members.* Full information about data collection is provided, school support team members fill in the Checklist about competence in materials and procedures (Supplementary Annex 2). School support team members organize an introductory visit to every school (experimental and control), where they • discuss planned activities and the necessary conditions (e.g., collecting of informed consent forms from parents, the need for computers for surveys, the need for a specific number of students, clarifying the participant coding system, making an agreement for its storage in accordance with research ethics, etc.); • inform/remind teachers to collect permission forms from parents for data collection; • arrange a time for the other three meetings with the parents of the experimental group.
(3). *Sharing training procedures and materials with school support team members* Full information (principles, agenda) about the teacher training is provided, school support team members fill in Table for keywords (School support team member) (Supplementary Annex 1) and Checklist about competence in materials and procedures (School support team member) (Supplementary Annex 2).
(4). *Meeting with parents* School support team members organize an introductory meeting with parents to establish contact and introduce the project. • During the introductory meeting, parents receive general information about the project as a whole, planned activities, and the opportunity to receive materials; give their agreement for participation and testing; and have the opportunity to answer questionnaires. • No materials are distributed there yet! The aim of the following meetings is to motivate parents in the experimental condition to participate in PROMEHS activities at home (using the student and parent handbooks) and share and discuss parenting practices in order to promote the mental health of their children.
(5). *Pre-test. Data collection in experimental and control schools* Paper-pencil or electronic data collection (students’, parents’, and teachers’ questionnaires). Student surveys are filled out in the presence of school support team members. Data collected from paper-pencil surveys must be filled into an online form (by a school support team member or researcher).
(6). *Training of teachers at the experimental schools* School support team member leads the 16 h training for teachers according to the agenda. Table for keywords (teacher) (Supplementary Annex 5) can be used as support material for teachers to help them become more familiar with the material. The evaluation will be done in written form at the very end of the teachers’ training and in a reflective cycle. See questionnaire in Questionnaire of evaluation of teacher training (Teacher) (Supplementary Annex 4). The aims of this evaluation are to: • receive feedback about the quality of the training in terms of usefulness; and • xmonitor teachers’ readiness to implement the PROMEHS program. Additionally, after school support team members collect filled-in questionnaires, two questions must be addressed in a reflective cycle: What have I achieved during the training? What questions remained unanswered? After the evaluation, the school support team member reviews the responses (both questionnaires and reflective cycle) with the national team and makes a general analysis of the training fidelity, acceptance of agenda, and teacher responsiveness, as well as any adaptations of the program. See questionnaire in Questionnaire of evaluation of teacher training (School support team member) (Supplementary Annex 3). Teachers are instructed to start their intervention immediately after the training for 12 weeks, with at least one activity per week. After the first activity in class, the student and parent handbooks are given to students. After each PROMEHS activity in class, we ask the teachers to review and reflect on their practice individually using the Teacher self-reflection form (Teacher) (Supplementary Annex 6). The teachers should prepare for a supervision by making written notes after each activity.
(7). Supervisions of teachers (3 × 3 h) in the experimental schools 1st supervision (approximately 2–3 weeks after teacher training), 2nd supervision (approximately 4–6 weeks after the 1st supervision), 3rd supervision (approximately 4–6 weeks after the 2nd supervision). • All supervisions have the same structure and content. • Additionally, the 3rd supervision includes the final evaluation. • Between supervisions, a support team member communicates with the school *via* e-mail or another platform. • During a supervision, the school support team member makes notes according to guidelines in Supervision summary (School support team member) (Supplementary Annex 7). Principles: Emotional support: “Thank you for your involvement.” Plan for (rules of) the meeting: “We have met to discuss the situation, answer questions, and plan the next activities. This is not about control.” Confidentiality: “Outside this group, each person can only share personal information with others,” “Let each participant express his/her opinion,” “Each person will have an opportunity to speak,” “Every participant is asked to speak from their own perspective,” “If there appear to be some problems, we will support each other and share responsibilities to find a solution for your school.”
Space for reflection: How do I feel? What is my attitude? What are my personal concerns? What resources do I have? During the supervision, the main questions are discussed together: Success. How did we succeed during this time? Challenges. What has been challenging? Adaptation. If changes were made to the program, what were they and why were they made? Continue to develop teachers’ understanding of the PROMEHS approach to the promotion of mental health by answering questions about the content of handbooks. During the supervision, the school support team member writes down specific observations on best practices and how the material has been adapted. After the supervision, a summary must be done. See Supervision summary (School support team member) (Supplementary Annex 7). During the 3rd supervision, the usual content is supplemented by an evaluation. Teachers are asked to fill in Final evaluation form in 3rd supervision (Teacher) (Supplementary Annex 8) and comment with questions of their own choice.
(8). *Meetings with school leaders of the experimental schools* Information for the administration about PROMEHS and how to support the intervention.
(9). *Meetings with parents of students of the experimental schools* Responsiveness evaluation of the parents and students. Parent survey (Parents) (Supplementary Annex 10).
(10). *Data collection for monitoring the quality of the implementation* If possible, the student survey should be carried out by school support team members among students who participated in the intervention. Use Student survey (Students) (Supplementary Annex 9) to evaluate how students felt and what the class environment was like. After the last supervision, a meeting with all school support team members should be organized (for a reflection on the process/about themselves). Work on the final report, including a brief summary of quantitative data from the student survey, and on finding out the best practices and cultural adaptations is also done at this point. As a result, a written report with specific initial recommendations should be developed • to improve the teachers’ training • to improve the handbooks • for educational policy
(11). *Post-test. Data collection in experimental and control schools* Paper-pencil or electronic data collection (students’, parents’, and teachers’ questionnaires). Student surveys are filled out in the presence of school support team members. Data collected from paper-pencil surveys must be filled into an online form. For support, it was recommended to use Checklist of activities (School support team member) (Supplementary Annex 11).

The essential components of the monitoring were selected following curriculum and research protocols and were described in the framework section. Materials and guidelines were available for the school support team, sharing information on data collection and training, meeting with school leaders, teacher training and supervisions, activities at schools, and parents’ meetings.

Aiming to evaluate the fidelity of the program’s implementation, data were collected after teacher training, during activities at school, and during supervisions from both school support team members and teachers. Measures included categorical scales (e.g., online, on-site, or mixed training) as well as continuous scales with a Likert-type scale (e.g., the question in Supplementary Annex 6 measuring the extent to which an activity from the handbook was implemented completely).

It was planned that dosage would be measured during the implementation: each teacher should have filled in a self-reflection form (Supplementary Annex 6) after each activity and taken it with him/her to their supervision. However, the actual number of the implemented activities was reported by teachers at the post-test stage. Considering that testing the PROMEHS program’s effectiveness took place in Europe during one of the waves of the COVID-19 pandemic, there were several threats to the filling-in of these forms. It can be assumed that some teachers gave up on implementing the program because of the stressful context of the COVID-19 pandemic and related epidemiological measures.

However, the sufficient variance of dosage, including significant deviations from the pre-planned length (min. 12 activities), provides the opportunity to test the dosage effect in relation to the effectiveness of the PROMEHS program.

The quality of implementation evaluates the skill and competence of the program providers according to the content and methods of the PROMEHS intervention manual. There were specific competence requirements for school support team members, and regular meetings related to testing, training, and supervisions were organized and reported. Several support materials were provided to strengthen their competence in PROMEHS materials (Supplementary Annex 1) and management of the field trial (Supplementary Annex 11).

The quality of evaluation addresses school support team members (self-reports) and teachers (self-reports and student reports). School support team members evaluated their own competence in teaching materials and procedures (Supplementary Annex 2) before starting on the implementation. Teachers evaluated their understanding of the task to be performed and their confidence in their ability to carry out this program, as well as their competence in the related teaching materials (Supplementary Annex 4). At the end of the intervention, students were asked to evaluate the manner in which the program was implemented (Supplementary Annex 9).

Considering the principles of the PROMEHS program emphasizing collaboration between school and family, teachers, students, and parents were all treated as participants, and their levels of responsiveness were measured. Teacher responsiveness was estimated after the teacher training and was evaluated by school support team members (Supplementary Annex 3). Students’ responsiveness was evaluated by teachers after each activity using a special self-reflection form (Supplementary Annex 6). Teacher responsiveness, according to support materials for their own mental health, was assessed during the last supervision (Supplementary Annex 8). Responsiveness measures were included in the post-test survey: students (aged nine and older) were asked to evaluate the usefulness of the student handbooks (Supplementary Annex 9), and parents were asked to evaluate the usefulness of parents’ meetings and the handbooks for parents and students as well (Supplementary Annex 10).

Adaptation refers to changes made to the original program during its implementation (program modification, reinvention). As a result, adaptation was integrated as an independent dimension with high value in the monitoring system in all stages of implementation, and qualitative data were collected. Adaptations were observed in several sources. Teachers filled out a self-reflection form (Supplementary Annex 6) after each activity and characterized what was changed and why, and they were also asked to describe their successes and any difficulties. This information gave a comprehensive picture of the adaptations made, reasons for these, the most successful practices, as well as activities where changes or updates would be welcomed. School support teams collected best practices and difficulties during supervisions (Supplementary Annex 7) and summarized them after the implementation to develop national-level recommendations for the implementation of the program.

## Discussion

A program can be evaluated as effective if it is implemented as intended. The fidelity of the intervention can be significantly increased by developing materials on content, what to implement, and the manner in which it should be implemented. The PROMEHS program filled this requirement by providing comprehensive, ready-to-use handbooks for teachers, students, families, and policymakers. Added value is related to the inclusion of content and process components in the monitoring system, where several materials can be used as tools to familiarize oneself with the content of the program while following the guidelines described in detailed procedures. Additionally, the monitoring measures include a checklist to ensure the implementation of all program components.

The PROMEHS monitoring system covers all the most relevant components of the intervention, starting with the development of the school support team, followed by teacher training, supervision, activities at schools, parents’ meetings, and student feedback. Specific requirements for the school support team members are described in the monitoring procedures to ensure quality. Moreover, these prepared the professional continuing education course for pedagogues so that PROMEHS could be maintained sustainably.

Providing support for people involved in the implementation of the program was recognized as a crucial principle, and therefore materials in the form of checklists were included to make the monitoring system user-friendly and helpful, allowing participants to practice self-monitoring during the implementation. A checklist on the content of handbooks allowed implementers to use it both as a training task and as a piece of evidence on how familiar both the school support team and teachers were with the provided materials. Considering that the usefulness and user-friendliness of tools can increase readiness to use monitoring tools, further research is needed on the applicability of the monitoring system after the project. It can be assumed that teacher self-report forms (e.g., Supplementary Annex 6) can be used to strengthen their self-reflection skills; however, further research is needed before confirming such a recommendation.

The implementation of the program is not always compatible with an aim to explore factors affecting its success or failure. A research strategy combining monitoring data and pre- post-test data allows the testing of a hypothesis about possible mediating or moderating effects of implementation characteristics on program outcomes. It can be assumed that diverse informants can evaluate different qualities of the program’s implementation, allowing key predictors of program efficacy and necessary support for program providers to be explored.

Both quantitative and qualitative (according to Dowling and Barry, 2020) data were collected for the assessment of fidelity and quality, responsiveness and dosage were measured quantitatively, and adaptation was evaluated exclusively using qualitative data. The reflections of teachers and observations of school support teams during supervisions provided an opportunity to explore nuanced and highly applicable experiences on how certain topics and activities were perceived in different countries, age groups, and backgrounds.

It is known that observational data are more reliable than self-reported data, and the reliability of measures can be strengthened by combining different data sources. It is important not to limit the monitoring only to activities in the main target group (students), since the intervention included activities focused on teachers, parents, and school-leaders as well. Direct observation was not included in the present monitoring system; however, this limitation was addressed by collecting multi-informant data from the program’s implementers (teachers), students, and their parents, as well as from the support team members, who provided teacher training and on-going supervisions and parents’ meetings. This strategy allows the implementation of the community engagement principle to be monitored as well, which is crucial to the sustainability of the program.

### Implications, limitations, and conclusion

The findings of this study highlight the importance of including several aspects often generally described as fidelity but which, nevertheless, allow the implementation process of a program to be evaluated from different angles, namely, dosage, responsiveness, quality, and adaptation.

This study also emphasizes the role of monitoring every aspect of implementation regarding both its content and its procedure. Moreover, it emphasizes the importance of building scientifically sound and, at the same time, user-friendly monitoring procedures in order not to overwhelm participants with data collecting but rather to support them during the implementation process. This study contributes to the field providing an elaborated framework for monitoring of implementation of different interventions. This supports both researchers and practitioners in developing, implementing, assessing, and sustaining the best possible practice in the intervention.

The strengths of this monitoring system are its observation of both content and process with scientifically sound dimensions, thus covering the whole spectrum of implementation, its collection of qualitative and quantitative data, and its use of a multi-informant approach. PROMEHS implementation during the COVID-19 pandemic allowed to document adaptation related with remote learning and computer mediated instructions.

The system also has some limitations. First, no direct observation of the teacher’s competence and interaction with students during the activities was available, limiting conclusions about the quality of the implementation of the program. Observation would be beneficial for providing more contextualized feedback and helping to develop teacher competence in instructing SEL. However, this can partly be offset with observations during supervisions when teachers interact with each other, which can also be used as an indicator of the manner in which they implement principles of the PROMEHS program. This limitation was partially neutralized by collecting evaluations from all groups of participants, direct observation of the responsiveness during teacher training was done by school support teams, whereas responsiveness of the students was evaluated by their parents. In the future, it would be useful to add direct observation during class activities to estimate quality of the implementation, as well as responsiveness of the students. Second, there was no monitoring of the control group. One critical point that was emphasized in the literature was the necessity to control other possible interventions in the control group. However, the COVID-19 pandemic context, with the related social distancing and remote learning, provided an opportunity to overcome this limitation since, due to the restrictions of the pandemic, the control group did not receive any alternative interventions. This naturally alleviated the necessity to monitor it.

## Data availability statement

The original contributions presented in this study are included in the article/Supplementary material, further inquiries can be directed to the corresponding author.

## Ethics statement

The studies involving human participants were reviewed and approved by the Ethics Committee for Humanities and Social Sciences Research Involving Human Participants of the University of Latvia on 12 December 2019. Written informed consent to participate in this study was provided by the participants or their legal guardian/next of kin.

## Author contributions

BM: lead writer, arranging the research in Latvia, contributing to the development of the monitoring system, and collecting data. IS: contributing to the development of the monitoring system and to writing. IG: a key contribution to designing the research and revising the manuscript. All authors contributed to the article and approved the submitted version.
