# Supplementary material for: Monitoring system of implementation of the Promoting Mental Health at Schools (PROMEHS) program
Source: Front Psychol. 2022 Oct 26;13:1043001. doi: 10.3389/fpsyg.2022.1043001 (PMC9645135; doi:10.3389/fpsyg.2022.1043001)
Supplement: Supplementary file 1 [file Data_Sheet_1.docx]

# Annexes

# Annex 1. Table for keywords (School support team)

Please read the teacher and student handbooks and write keywords to help you recall the content of each lesson.

| Theme/topic  goal/activity | Keywords |
| --- | --- |
| 1.1.1.1. |  |
| 1.1.1.2. |  |
| 1.1.2.1. |  |
| 1.1.2.2. |  |
| 1.1.3.1. |  |
| 1.1.3.2. |  |
| 1.2.1.1. |  |
| 1.2.1.2. |  |
| 1.2.2.1. |  |
| 1.2.2.2. |  |
| 1.2.3.1. |  |
| 1.2.3.2. |  |
| 1.3.1.1. |  |
| 1.3.1.2. |  |
| 1.3.2.1. |  |
| 1.3.2.2. |  |
| 1.4.1.1. |  |
| 1.4.1.2. |  |
| 1.4.2.1. |  |
| 1.4.2.2. |  |
| 1.4.3.1. |  |
| 1.4.3.2. |  |
| 1.4.4.1. |  |
| 1.4.4.2. |  |
| 1.4.5.1. |  |
| 1.4.5.2. |  |
| 1.5.1.1. |  |
| 1.5.1.2. |  |
| 1.5.2.1. |  |
| 1.5.2.2. |  |
| 2.1.1.1. |  |
| 2.1.1.2. |  |
| 2.1.2.1. |  |
| 2.1.2.2. |  |
| 2.1.3.1. |  |
| 2.1.3.2. |  |
| 2.1.4.1. |  |
| 2.1.4.2. |  |
| 2.1.5.1. |  |
| 2.1.5.2. |  |
| 2.2.1.1. |  |
| 2.2.1.2. |  |
| 2.2.2.1. |  |
| 2.2.2.2. |  |
| 3.1.1.1. |  |
| 3.1.1.2. |  |
| 3.1.2.1. |  |
| 3.1.2.2. |  |
| 3.1.3.1. |  |
| 3.1.3.2. |  |
| 3.1.4.1. |  |
| 3.1.4.2. |  |
| 3.2.1.1. |  |
| 3.2.1.2. |  |
| 3.2.2.1. |  |
| 3.2.2.2. |  |
| 3.3.1.1. |  |
| 3.3.1.2. |  |
| 3.3.2.1. |  |
| 3.3.2.2. |  |
| 3.3.3.1. |  |
| 3.3.3.2. |  |
| 3.3.4.1. |  |
| 3.3.4.2. |  |
| 3.3.5.1. |  |
| 3.3.5.2. |  |
| 3.3.6.1. |  |
| 3.3.6.2. |  |

Annex 2. Checklist about competence in materials and procedures (School support team)

Please evaluate your readiness to implement the project in practice.

| Statement | Completely disagree | |  | Completely agree | |
| --- | --- | --- | --- | --- | --- |
| 1. I am confident about the steps of data collection. | 1 | 2 | 3 | 4 | 5 |
| 2. I know the contents of the teacher and student handbooks well. | 1 | 2 | 3 | 4 | 5 |
| 3. I am ready to build a partnership with school leaders to implement PROMEHS. | 1 | 2 | 3 | 4 | 5 |
| 4. I am competent to present PROMEHS to parents. | 1 | 2 | 3 | 4 | 5 |
| 5. I am competent to lead teachers’ training. | 1 | 2 | 3 | 4 | 5 |
| 6. I am competent to supervise teachers. | 1 | 2 | 3 | 4 | 5 |

Annex 3. Questionnaire of evaluation of teacher training (School support team)

*Name, surname _________________________________________*

*School _____________________________________________________*

*Date _________________ Time (from, to) __________________________*

*Duration (contact hours) _________ Number of participants __________*

*PLACE: ___ONLINE, ___ON-SITE, ___MIXED*
*FORM: ___LECTURE, ___LECTURE + INTERACTION, ___VIDEO ONLY*

Please evaluate the implementation of the teacher training agenda.

| Criteria | Very poor | Poor | Ok | Good | Very good | Excellent |
| --- | --- | --- | --- | --- | --- | --- |
| 1. Implementation of agenda (if compared with planned agenda) | 1 | 2 | 3 | 4 | 5 | 6 |
| 2. Acceptance of content | 1 | 2 | 3 | 4 | 5 | 6 |
| 3. Teachers’ responsiveness | 1 | 2 | 3 | 4 | 5 | 6 |
| 4. If there were any obstacles or changes in teacher training, please describe them:  QUALITATIVE DESCRIPTION OF THE CHANGES IN THE TEACHER TRAINING NOT MENTIONED SO FAR | | | | | | |

Annex 4. Questionnaire of evaluation of teacher training (Teacher)

*School ______________________________________________________*

*Date _______________ Time (from, to) ____________________________*

Please evaluate your readiness to implement PROMEHS at school.

| Statement | Very poor | Poor | Ok | Good | Very good |
| --- | --- | --- | --- | --- | --- |
| 1. Usefulness of training | 1 | 2 | 3 | 4 | 5 |
| 2. Sufficiency of information | 1 | 2 | 3 | 4 | 5 |
| 3. Understanding of the task to be performed | 1 | 2 | 3 | 4 | 5 |
| 4. Understanding of the importance of the promotion of mental health at school | 1 | 2 | 3 | 4 | 5 |
| 5. Confidence in your ability to carry out the program | 1 | 2 | 3 | 4 | 5 |
| 6. Your comments and questions. | | | | | |

Annex 5. Table for keywords (Teacher)

Please read the teacher handbook and write keywords to help you recall the content of each lesson.

| Theme/topic  goal/activity | Keywords |
| --- | --- |
| 1.1.1.1. |  |
| 1.1.1.2. |  |
| 1.1.2.1. |  |
| 1.1.2.2. |  |
| 1.1.3.1. |  |
| 1.1.3.2. |  |
| 1.2.1.1. |  |
| 1.2.1.2. |  |
| 1.2.2.1. |  |
| 1.2.2.2. |  |
| 1.2.3.1. |  |
| 1.2.3.2. |  |
| 1.3.1.1. |  |
| 1.3.1.2. |  |
| 1.3.2.1. |  |
| 1.3.2.2. |  |
| 1.4.1.1. |  |
| 1.4.1.2. |  |
| 1.4.2.1. |  |
| 1.4.2.2. |  |
| 1.4.3.1. |  |
| 1.4.3.2. |  |
| 1.4.4.1. |  |
| 1.4.4.2. |  |
| 1.4.5.1. |  |
| 1.4.5.2. |  |
| 1.5.1.1. |  |
| 1.5.1.2. |  |
| 1.5.2.1. |  |
| 1.5.2.2. |  |
| 2.1.1.1. |  |
| 2.1.1.2. |  |
| 2.1.2.1. |  |
| 2.1.2.2. |  |
| 2.1.3.1. |  |
| 2.1.3.2. |  |
| 2.1.4.1. |  |
| 2.1.4.2. |  |
| 2.1.5.1. |  |
| 2.1.5.2. |  |
| 2.2.1.1. |  |
| 2.2.1.2. |  |
| 2.2.2.1. |  |
| 2.2.2.2. |  |
| 3.1.1.1. |  |
| 3.1.1.2. |  |
| 3.1.2.1. |  |
| 3.1.2.2. |  |
| 3.1.3.1. |  |
| 3.1.3.2. |  |
| 3.1.4.1. |  |
| 3.1.4.2. |  |
| 3.2.1.1. |  |
| 3.2.1.2. |  |
| 3.2.2.1. |  |
| 3.2.2.2. |  |
| 3.3.1.1. |  |
| 3.3.1.2. |  |
| 3.3.2.1. |  |
| 3.3.2.2. |  |
| 3.3.3.1. |  |
| 3.3.3.2. |  |
| 3.3.4.1. |  |
| 3.3.4.2. |  |
| 3.3.5.1. |  |
| 3.3.5.2. |  |
| 3.3.6.1. |  |
| 3.3.6.2. |  |

Annex 6. Teacher self-reflection form (Teacher)

*Name, surname_________________________________School____________________________________* Date (-s): _______________________

Please evaluate the lesson material and reflect on your lesson experience immediately after the lesson.

| Lesson number: ____ ____ ____ ____ (theme, topic, goal, activity) | Strongly disagree | Disagree | Un-decided | Agree | Strongly agree |
| --- | --- | --- | --- | --- | --- |
| 1. The activity from the handbook was implemented completely. | 1 | 2 | 3 | 4 | 5 |
| If not, what was changed and why? | | | | | |
| 2. The topic of the lesson was relevant to my students. | 1 | 2 | 3 | 4 | 5 |
| 3. Students actively participated in the lesson | 1 | 2 | 3 | 4 | 5 |
| 4. Students shared their opinion. | 1 | 2 | 3 | 4 | 5 |
| 5. Why I chose this topic for the lesson. | | | | | |
| 6. What was successful in the lesson? What difficulties did I meet? | | | | | |

Annex 7. Supervision summary (School support team member)

*Name, surname___________________________________School__________________________________ Supervision number: 1 2 3*

*Date ___________________Duration (from to) _____________Number of participants _________*

*Number of participants who prepared a written self-reflection before the supervision __________*

| *Best practices:* |
| --- |
| *What was changed and why:* |

Annex 8. Final evaluation form in 3^rd^ supervision (Teacher)

*Name, surname________________________________School_______________________________*

Please answer these questions about the PROMEHS program.

| 1. Please provide specific examples of how you observed the development of your students’ social-emotional skills. | | | | | | |
| --- | --- | --- | --- | --- | --- | --- |
| 1. Please provide specific examples of what changes you observed in your teaching practice | | | | | | |
| 1. How useful was the PROMEHS handbook for the development of your own mental health and resilience? | 5  Extremely useful | 4  Very useful | 3  Useful | 2  Slightly useful | 1  Not useful at all | 0  I didn’t use it. |
| 1. What are your personal gains from the project? | | | | | | |
| 1. How will you sustain the PROMEHS approach to the promotion of mental health? What are the next steps to continue this work? | | | | | | |

Annex 9. Student survey (Students)

*Date _____________ School_________________________________________________ Grade ____________*

*Your age _______________ Gender: male/female (boy/girl)*

Please evaluate the PROMEHS lessons.

| Statement | Strongly disagree | Disagree | Undecided | Agree | Strongly agree |
| --- | --- | --- | --- | --- | --- |
| 1. I enjoyed these lessons. | 1 | 2 | 3 | 4 | 5 |
| 1. The topics of the lessons were important to me. | 1 | 2 | 3 | 4 | 5 |
| 1. I had the possibility to express my opinion during the lessons. | 1 | 2 | 3 | 4 | 5 |
| 1. I used the student handbook at home. | 1 | 2 | 3 | 4 | 5 |
| 1. The activities in the student handbook were interesting. | 1 | 2 | 3 | 4 | 5 |

Annex 10. Parent survey (Parents)

*Date ________________________________ School_______________________________________________________________________*

*Child’s grade _______________ Child’s age __________________ Child’s gender: male/female*

Please evaluate the PROMEHS project’s activities.

| Questions | 0  I didn’t participate / Don’t know | 1  Not useful at all | 2  Slightly useful | 3  Useful | 4  Very useful | 5  Extremely useful |
| --- | --- | --- | --- | --- | --- | --- |
| 1. How useful were the parents’ meetings? | 0 | 1 | 2 | 3 | 4 | 5 |
| 1. How useful was the handbook for parents? | 0 | 1 | 2 | 3 | 4 | 5 |
| 1. How useful was the handbook for students? | 0 | 1 | 2 | 3 | 4 | 5 |
| 1. Have you noticed any emotional or behavioral changes in your child as a result of their PROMEHS lessons? | No | Yes |  |  | |  |
| If yes, please describe these changes: | | | | | | |
| 1. Please write what you will continue to do that you have learned during this project. | | | | | | |

Annex 11. Checklist of activities (School support team)

*Name of school support team member____________________________________________*

*School___________________________________________________*

Check the things that you have done with √.

| Activity | Done |
| --- | --- |
| Before the intervention |  |
| Read the teacher and student handbooks |  |
| Fill in the table with keyword (Annex 1) |  |
| Clarify the principles of data coding |  |
| Fill in the checklist about competence in materials and procedures (Annex 2) |  |
| Meeting with school leaders |  |
| Meeting with parents:   - Agreed time and place with the school - Prepared materials for the meeting - Obtained informed agreements from parents |  |
| Pre-test:   - Questionnaires (students, teachers, parents) prepared for the distribution - Lists of students are kept in accordance with the ethics of the study - Filled in data with an electronic survey |  |
| During the intervention |  |
| Teacher training:   - Time and place agreed with the school - Prepared materials for training (agenda, handbooks, presentation, etc.) - Coffee break and lunch - Questionnaire of evaluation of teacher training (school support team member) (Annex 3) - Questionnaire of evaluation of teacher training (teachers) (Annex 4) - Teacher self-reflection form (Annex 6) - Table of keywords (teacher) (Annex 5) - Report about teacher training |  |
| First supervision:   - Time and place agreed with the school - Prepared materials for supervision - Teacher self-reflection form (1^st^ supervision) (Annex 6) - Supervision summary (1^st^ supervision) (Annex 7) - Report of the 1^st^ supervision - Teacher self-reflection forms are kept in accordance with the ethics of the study. |  |
| Second supervision:   - Time and place agreed with the school - Prepared materials for supervision - Teacher self-reflection form (2^nd^ supervision) (Annex 6) - Supervision summary (2^nd^ supervision) (Annex 7) - Report of the 2^nd^ supervision - Teacher self-reflection forms are kept in accordance with the ethics of the study. |  |
| Third supervision:   - Time and place agreed with the school - Prepared materials for supervision - Teacher self-reflection form (3^rd^ supervision) (Annex 6) - Supervision summary (3^rd^ supervision) (Annex 7) - Final evaluation form in 3^rd^ supervision by teachers (Annex 8) - Report of the 3^rd^ supervision - Teacher self-reflection forms and final evaluations are kept in accordance with the ethics of the study. |  |
| After the intervention |  |
| Post-test questionnaires (students, teachers) |  |
| Feedback from students about PROMEHS lessons (Annex 9) |  |
| Meeting with parents:   - Time and place agreed with the school - Prepared materials for the parent survey (Annex 10) |  |
| Meeting with national school support team to discuss and develop the final report |  |
| Implementation of the program at the schools in the control group |  |
